# Collegiate Male Soccer Players Reporting Their Perceived Exertion: Differences in Internal and External Intensity between Different RPE Values and Zones

**DOI:** 10.5114/jhk/209543

**Published:** 2025-11-19

**Authors:** Zheng Li, Shenglei Qin, Xiaotian Li, Dingmeng Ren

**Affiliations:** 1 China Football College, Beijing Sport University, Beijing, China.; 2 APAC Team, Catapult Sports, Melbourne, Australia.; 3 Physical Education Department, Central South University, Changsha, China.; 4 School of Sport Training, Wuhan Sports University, Wuhan, China.

**Keywords:** heart rate, load, training, RPE, team sports, distance covered

## Abstract

This study aimed to: (1) investigate the association between subjective perception (RPE, sRPE) and objective metrics (TRIMP, average HR and peak HR both expressed as %HR_max_, total distance, PlayerLoad^TM^, acceleration distance > 1 m/s^2^, and running distance ≥14 km/h) in training monitoring using a repeated-measure design, and (2) assess the utility of RPE levels (values and zones) in differentiating among particular training intensities. This study used a longitudinal study design. Fifteen collegiate male soccer players (age 19.6 ± 0.8 years, body height 179.1 ± 5.4 cm, body mass 70.8 ± 4.9 kg, BMI 22.1 ± 1.7 kg/m^2^, body fat rate 10.9 ± 2.3%, HR_rest_ 56.6 ± 8.0, HR_max_ 194.9 ± 7.3) participated in the study. GPS-based wearable devices and the RPE scale were used to training monitoring. Based on 676 observations from 69 training sessions, a moderate to large correlation was found between the SRPE and internal/external loads (r ranges from 0.34 to 0.60, p < 0.001), while a small to moderate correlation was observed between the RPE and internal/external intensity (r ranges from 0.11 to 0.35, p < 0.001). When distributing training intensity, significant effects were found between RPE values and RPE zones (p < 0.05). Our study provides evidence for understanding the relationship between the subjective load assessment method (RPE and sRPE) and objective methods. Specific ranges of internal and external intensity variables can be divided between RPE value (1–9 AU) and the RPE zone (low, moderate, high).

## Introduction

Monitoring training loads is critical to improving performance, adjusting training plans, assessing athletes’ recovery needs, and minimizing the risk of non-functional overreaching, injury, and illness (Bourdon et al., 2017; Chiu et al., 2024). The load is generally divided into two dimensions for evaluation, i.e., the internal load and the external load (Vanrenterghem et al., 2017). In team sports training and competitions, a greater external load increases metabolic energy cost and soft tissue force absorption/production, thereby increasing the internal load (McLaren et al., 2018). The relationship between external and internal loads reflects the dose-response nature of the applied training stimulus and the athlete’s internal response (Akubat et al., 2014; Bartlett et al., 2017; Scanlan et al., 2014).

In training practice, the external load focuses primarily on the objective work athletes have completed during training (Fox et al., 2018a; Gonçalves et al., 2025). In running-based team sports such as soccer, indicators such as total distance and distance covered at different velocity thresholds, acceleration, deceleration, and instantaneous acceleration accumulation are commonly used to evaluate external loads (Asian- Clemente et al., 2025; Beato et al., 2018; Hennessy and Jeffreys, 2018; Theodoropoulos et al., 2020). However, the stimulus for training-induced adaptations is the relative physiological stress (internal load) exerted on the athlete, not the external load (Impellizzeri et al., 2004). The relationship between internal and external loads during training and competition is also influenced by the players’ performance level, the type of training implemented, and the phase of the competitive season (De Dios-Álvarez et al., 2025; Douchet et al., 2024; Savolainen et al., 2025). Therefore, by monitoring the training process, understanding the relationship between internal and external training loads can potentially improve training prescription, periodization, and athletes’ management (Bartlett et al., 2017; Burgess, 2017; Castillo et al., 2016; McLaren et al., 2018).

Compared with semi-invasive assessment methods such as cardiac output and oxygen uptake (Vanrenterghem et al., 2017), the subjective rating of perceived exertion (RPE) scale (Borg, 1982) offers distinct practical advantages. Furthermore, its convenient acquisition method and straightforward training load monitoring contribute to its widespread adoption in training practice. The session-RPE (sRPE) is obtained through “RPE × Duration (min)” (Foster et al., 2001); the RPE here evaluates the entire training process rather than an instantaneous rating (Foster et al., 1995). A large body of research has demonstrated the relationship between the sRPE and objective internal and external loads, showing a moderate to very large correlation with total distance, training impulse (TRIMP), high-speed running distance and the accelerometer-derived load (McLaren et al., 2018). Moreover, the high correlation between the sRPE and TRIMP, a heart rate (HR) based internal load, proves that it is an effective method for assessing the internal load (Casamichana et al., 2013; Impellizzeri et al., 2004; Kelly et al., 2016; Rodriguez-Marroyo and Antonan, 2015).

However, notably, most prior studies have predominantly applied Pearson product-moment correlation analysis. In this case, errors between observations are considered independent (Cohen et al., 2015). In athletic training research, longitudinal designs inherently involve repeated measurements from individual athletes across multiple training sessions. This collection of data from athletes across multiple training sessions, where a single athlete provides multiple data points, appears to violate the assumption of independence (Bakdash and Marusich, 2017; McLaren et al., 2018). Averaging multiple measurements of an athlete before correlation analysis may solve the problem of data independence, but may produce misleading results if meaningful individual differences exist (Bakdash and Marusich, 2017). To resolve this methodological dilemma, biostatistical techniques originally developed for analyzing paired repeated measurements (Bland and Altman, 1995a, 1995b) have been systematically adapted by Bakdash and Marusich (2017) through their repeated measures correlation (rmcorr) framework. Rmcorr adjusts for inter-individual variation via analysis of covariance. By removing the between-individual variance, a best linear fit line with the same slope but different intercepts was provided for each participant (Bakdash and Marusich, 2017).

While the session-RPE (sRPE) demonstrates convergent validity with both internal (TRIMP: r = 0.57–0.77) and external load metrics (total distance: r = 0.80; PlayerLoad^TM^: r = 0.74–0.84) (Casamichana et al., 2013; Scott et al., 2013), its prescriptive utility as an intensity index shows weaker associations. Specifically, the RPE exhibits only moderate correlations with exercise intensity biomarkers including the average HR (%HR_max_) (r = 0.12–0.29) (Rago et al., 2022; Wilke et al., 2016) and the peak HR (%HR_max_) (r = 0.23) (Rago et al., 2022). When considering the relationship between the RPE and HR intensity indicators, different RPE values should correspond to different average HRs (%HR_max_) (Costa et al., 2022). In the study by Costa et al. (2022) on the training load of professional female soccer players over six weeks, the analysis revealed a quasi-linear dose-response association between the RPE and the average HR (%HR_max_). Median average HR (%HR_max_) values progressively rose with RPE increments (2–5), median (interquartile range) as follows: RPE 2 = 68% (65–69%), RPE 3 = 72% (69–75%), RPE 4 = 79% (76–82%), and RPE 5 = 84% (81–87%). However, this only considers the RPE in the range of 2–5, and research is needed to expand the RPE range. Another study on professional ice hockey players showed that within the RPE range of 2–7, there were significant differences in HR intensity indicators (average HR [%HR_max_], peak HR [%HR_max_]) between partial RPE values (Rago et al., 2022). Not all pairwise comparisons in the study by Rago et al. (2022) revealed statistically significant differences. In an earlier study (Seiler and Kjerland, 2006), researchers divided RPE intensity into three intervals based on the first and second ventilatory thresholds of endurance athletes, zone 1: 1 ≤ RPE ≤ 4, zone 2: 5 ≤ RPE ≤ 6 and 7 ≤ RPE ≤ 10. Lovell et al. (2013) also adopted the same range in the rugby intensity classification, respectively described as low, moderate and high. At present, when using the RPE to divide training intensity, research on a larger RPE range is needed, and considering whether the RPE can capture the internal and external intensity changes corresponding to each score in detail, it is also necessary to consider partitioning the RPE as low, moderate and high.

In this context, this study incorporated internal load metrics (TRIMP) and intensity metrics (average HR and peak HR both expressed as %HR_max_). External load metrics included total distance, PlayerLoad^TM^, acceleration distance > 1 m/s^2^ and running distance ≥14 km/h, each metric was time-normalized, with the resultant per-minute values representing intensity variables. Based on previous studies on the within-subject correlation of the RPE and its application in categorizing training intensity (Bakdash and Marusich, 2017; Costa et al., 2022; Rago et al., 2022), our first objective was to examine the relationship between subjective methods (sRPE and RPE) and internal/external variables using the rmcorr approach in collegiate male soccer players. The second objective was to assess the utility of RPE levels (values and zones) in differentiating training intensity.

## Methods

### Participants

Fifteen collegiate male soccer players (age 19.6 ± 0.8 years, body height 179.1 ± 5.4 cm, body mass 70.8 ± 4.9 kg, body fat rate 10.9 ± 2.3 %, HR_rest_ 56.6 ± 8.0 bpm, HR_max_ 194.9 ± 7.3 bpm) participated in the study. All participants were nationally certified first-class athletes or higher, with over 10 years of systematic training experience and current participation in the highest collegiate division. Participants were fully informed of potential risks and benefits associated with data collection and advised that they could withdraw at any time without any consequences. We obtained written informed consent from all athletes and their legal guardians. All experimental procedures complied with the Declaration of Helsinki, and were approved by the ethics committee of the Wuhan Sports University, Wuhan, China (protocol code: 2023070; approval date: 28 September 2023).

### Procedure

Prior to data collection, we measured the players' baseline information, including body height, body mass, and the body fat content, using the InBody 770 (InBody 770, South Korea).

### Resting Heart Rate Test

To obtain the resting heart rate, we used the Polar Team Pro (Polar Team Pro, Finland) to monitor the players' heart rates while they were seated indoors. The day before the test, we informed athletes to avoid intense exercise, caffeine, or other stimulants. Participants were required to remain awake and quiet throughout the process, and the average heart rate over a 5-min period was recorded as the resting heart rate.

### Maximal Heart Rate Test

To determine the athletes' maximal heart rate, we used the 30–15 intermittent fitness test (30–15 IFT), which has been demonstrated to have good validity and reliability in assessing aerobic capacity (Buchheit et al., 2011; Čović et al., 2016). We conducted this test on a natural grass soccer pitch. Players ran to the designated area following an audio cue, performing 30-s shuttle runs interspersed with 15 s of walking recovery. The initial speed was 8 km/h (for the first 30-s shuttle run), and the speed increased by 0.5 km/h every 30 s thereafter. Athletes were considered eliminated if they failed to reach the designated point in sync with the audio cue three consecutive times. The maximal heart rate during the test was recorded.

### Training Monitoring

We used the Catapult Vector S7 (Catapult Vector S7, Catapult, Australia) wearable device to measure internal and external loads during training. In running-based team sports such as soccer, global positioning system (GPS)-based monitoring systems have proven to be effective means of detecting players’ activity levels (Duffield et al., 2010; Johnston et al., 2013). The device monitors satellite GPS positioning (10-Hz GPS), local positioning information, inertial sensors, and heart rate data. Players wore a vest that secured the device at the mid-scapular region of the back. Data were processed using OpenField software provided by the Catapult company.

The external training load was represented by total distance (Total Dis, m), total PlayerLoad^TM^ (Total PL, AU), distance > 14 km/h (Dis > 14 km/h, m), and acceleration distance > 1 m/s^2^ (Acc Dis, m). The above metrics were divided by the number of minutes to obtain training intensity. PlayerLoad^TM^ (PL) was derived using an enhanced vector magnitude algorithm based on the accelerometer data. It was calculated as the sum of the squared instantaneous rates of change in acceleration across three orthogonal planes, divided by 100 (Boyd et al., 2011).


$${\text{PlayerLoad}}^{\text{TM}}=\frac{\sqrt{\left({\left({\text{a}}_{\text{x1}}-{\text{a}}_{\text{x}-\text{1}}\right)}^{2}+{\left({\text{a}}_{\text{y}1}-{\text{a}}_{\text{y}-1}\right)}^{2}+{\left({\text{a}}_{\text{z}1}-{\text{a}}_{\text{z}-1}\right)}^{2}\right)}}{100}$$

where a_x_ = mediolateral accelerometer; a_y_ = anteroposterior accelerometer; a_z_ = vertical accelerometer. PlayerLoad^TM^ per minute (AU/min) is commonly used as a measure of movement intensity across a range of physical activities (Cormack et al., 2013; Fox et al., 2018b; Mooney et al., 2013).

The internal training load was measured using the sRPE and TRIMP method proposed by Banister (1991) (TRIMP1) and Edwards (1993) (TRIMP2), calculated as follows:

sRPE = T × RPE


$$\text{TRIMP1}=\text{T ×}\frac{\left({\text{HR}}_{\text{ex}}-{\text{HR}}_{\text{rest}}\right)}{\left({\text{HR}}_{\max}-{\text{HR}}_{\text{rest}}\right)}\times 0.64^{1.92\frac{\left({\text{HR}}_{\text{ex}}-{\text{HR}}_{\text{rest}}\right)}{\left({\text{HR}}_{\max}-{\text{HR}}_{\text{rest}}\right)}}$$

**TRIMP2 = (time in 50–60 %HR_max_) × 1 + (time in 60–70 %HR_max_) × 2 + (time in 70–80 %HR_max_) × 3 + (time in 80–90 %HR_max_) × 4 + (time 90–100 in %HR_max_) × 5**

where T expresses the duration (min) of the session, the HR_ex_ is the average HR during a session, HR_rest_ is the average heart rate during rest, and HR_max_ is the maximum HR during the 30–15 intermittent fitness test. The “time” in the TRIMP2 calculation is the duration (min) spent in the relevant zone of %HR_max_.

Based on previous studies (Costa et al., 2022; Rago et al., 2022), internal training intensity was represented by the average HR (%HR_max_) and the peak HR (%HR_max_).

Before each training session, the devices were activated and assigned to the corresponding players. The start and end times of training (in minutes) were recorded, excluding post-training recovery sessions. RPE values, obtained through the CR-10 scale (Foster et al., 2001), were collected individually from players within 30 minutes after each training session. To minimize external influences, players were not informed of their teammates’ or coaches’ RPE values. If a player missed a full training session due to injury, competition, or unforeseen circumstances, their data were excluded from the study. We performed data cleaning to exclude fragmented or missing data caused by uncontrollable factors, ensuring only reliable data were analyzed.

### Statistical Analysis

To characterize the inter-individual and intra-individual variability of variables, the coefficient of variation (CV) was calculated by dividing the standard deviation (SD) by the mean and then multiplying by 100%. According to scholars' recommendations (Hopkins et al., 2009; Hopkins, 2004) and practices in the field of sports training (Haugen and Buchheit, 2016), the smallest worthwhile change (SWC) was calculated by multiplying the inter-individual CV by 0.3. We used the repeated measures intraclass correlation (rmcorr) to assess the validity of the sRPE. Rmcorr utilizes analysis of covariance (ANCOVA) to statistically adjust for inter-individual variability and account for the non-independence of observations. By removing between-participant variance in measurements, rmcorr provides the best linear fit for each participant using parallel regression lines with the same slope but different intercepts (Bakdash and Marusich, 2017). The magnitude of correlation was qualitatively interpreted using the following criteria: trivial (r ≤ 0.1), small (r = 0.1–0.3), moderate (r = 0.3–0.5), large (r = 0.5–0.7), very large (r = 0.7–0.9), and almost perfect (r ≥ 0.9) (Hopkins et al., 2009).

The mixed-effects model was used to examine the relationship between two RPE intensity classification methods (values and zones) and intensity variables. The RPEs (values and zones) were treated as fixed effects, subjects as random effects, and intensity variables as dependent variables. When significant effects were identified, Bonferroni tests were used for pairwise comparisons. The *t*-statistics derived from the mixed model were converted into effect size correlations (r) and interpreted as previously described (Rago et al., 2022). All analyses met the assumptions of linearity, homoscedasticity, and approximate normality.

Descriptive statistics were expressed as Mean ± SD, with statistical significance set at *p* < 0.05. Data analysis was conducted using RStudio (Version 2024.09.1+394, RStudio, PBC, Boston, MA.) software, the main R packages used were rmcorr (Bakdash and Marusich, 2017) and lme4 (Bates et al., 2015).

## Results

The data included 676 observations generated by 69 training sessions from September 2023 to May 2024 and the data of the RPE = 10 were not included due to the small number of observations (n = 6).

Table 1 presents the descriptive data, individual variability, and smallest worthwhile change of training loads and intensity.

**Table 1:** Descriptive values and individual variability of training load and intensity (n = 676 individual observations).

	Mean ± SD	Interindividual variability (%)	Intraindividual variability (%)	SWC
sRPE (AU)	605.78 ± 240.08	39.63 (38.11–41.1)	34.09 (30.26–37.91)	72.02
TRIMP1 (AU)	101.15 ± 38.84	38.4 (36.93–39.82)	30.81 (29.37–32.25)	11.65
TRIMP2 (AU)	211.28 ± 68.54	32.44 (31.19–33.64)	29.47 (28.00–30.94)	20.56
Total Dis (m)	6397.60 ± 1659.73	25.94 (24.95–26.9)	24.47 (23.01–25.93)	497.92
Total PL (AU)	719.66 ± 190.77	26.51 (25.49–27.49)	23.2 (21.82–24.59)	57.23
Acc Dis > 1 m/s^2^ (m)	1100.16 ± 416.08	37.82 (36.37–39.22)	35.09 (33.71–36.46)	124.82
Dis > 14 km/h (m)	1008.86 ± 730.91	72.45 (69.67–75.13)	71.90 (68.94–74.86)	219.27
RPE (AU)	5.59 ± 1.85	33.04 (31.77–34.26)	25.67 (21.49–29.86)	0.55
Avg HR (%HR_max_)	65.25 ± 5.84	8.94 (8.6–9.27)	7.87 (7.39–8.36)	1.75
Peak HR (%HR_max_)	89.64 ± 6.56	6.21 (5.97–6.44)	5.35 (4.77–5.94)	1.67
Total Dis/min (m/min)	60.40 ± 14.28	23.65 (22.74–24.53)	22.23 (21.47–22.99)	4.29
Total PL/min (AU/min)	6.77 ± 1.57	23.16 (22.27–24.01)	19.47 (18.75–20.19)	0.47
Acc Dis > 1 m/s^2^ /min (m/min)	10.37 ± 3.87	37.29 (35.86–38.67)	34.58 (32.74–36.42)	1.16
Dis > 14 km/h/min (m/min)	9.50 ± 6.88	72.39 (69.60–75.06)	71.51 (67.85–75.17)	2.06

SWC: smallest worthwhile change; sRPE: session rating of perceived exertion; TRIMP1: training impulse, heart rate based internal load algorithm by Banister (1991); TRIMP2: heart rate based internal load algorithm by Edwards (1993); Total Dis: total running distance; Total PL: total PlayerLoad^TM^; Acc Dis > 1 m/s^2^: accelerate distance during acceleration threshold > 1 m/s^2^; Dis > 14 km/h: running distance during speed threshold > 14 km/h; Avg HR (%HR_max_): Average heart rate expressed as a percentage of the maximum heart rate; Peak HR (%HR_max_): Peak heart rate expressed as a percentage of the maximum heart rate

Table 2 presents the correlation between the sRPE and training load/intensity. The results indicated that the sRPE exhibited moderate to strong correlations with both internal and external load variables, with correlation coefficients (r) ranging from 0.34 to 0.60 (*p* < 0.001). In contrast, the RPE demonstrated weaker correlations with intensity metrics, exhibiting only small to moderate associations, with correlation coefficients (r) ranging from 0.11 to 0.35 (*p* < 0.01).

**Table 2:** Relationship between subjective and objective methods of training load and intensity

Load (sRPE)	r	95%CIs	Descriptor	*p*
TRIMP1 (AU)	0.56	0.51 to o.61	Large	< 0.001
TRIMP2 (AU)	0.60	0.55 to 0.65	Large	< 0.001
Total Dis (m)	0.48	0.42 to 0.54	Moderate	< 0.001
Total PL (AU)	0.56	0.51 to 0.61	Large	< 0.001
Acc Dis > 1 m/s^2^ (m)	0.34	0.27 to 0.40	Moderate	< 0.001
Dis > 14km/h (m)	0.35	0.28 to 0.41	Moderate	< 0.001
Intensity (RPE)				
Avg HR (%HR_max_)	0.32	0.25 to 0.39	Moderate	< 0.001
Peak HR (%HR_max_)	0.35	0.28 to 0.42	Moderate	< 0.001
Total Dis/min (m/min)	0.11	0.03 to 0.18	Small	0.005
Total PL/min (AU/min)	0.19	0.12 to 0.26	Small	< 0.001
Acc Dis > 1 m/s^2^/min (m/min)	0.12	0.04 to 0.19	Small	0.003
Dis > 14 km/h/min (m)	0.25	0.17 to 0.32	Small	< 0.001

sRPE: session rating of perceived exertion; TRIMP1: training impulse, heart rate based internal load algorithm by Banister (1991); TRIMP2: heart rate based internal load algorithm by Edwards (1993); Total Dis: total running distance; Total PL: total PlayerLoad^TM^; Acc Dis > 1 m/s^2^: accelerate distance during acceleration threshold > 1 m/s^2^; Dis > 14 km/h: running distance during speed threshold > 14 km/h; Avg HR (%HR_max_): average heart rate expressed as a percentage of the maximum heart rate; Peak HR (%HR_max_): peak heart rate expressed as a percentage of the maximum heart rate

**Figure 1:**
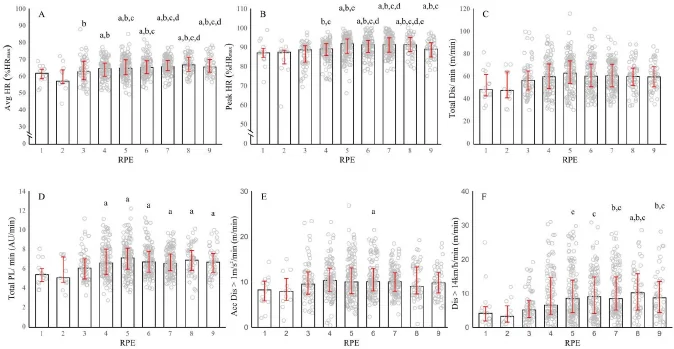
Distribution of (A) average HR (%HR_max_), (B) peak HR (%HR_max_), (C) total distance/min, (D) total PlayerLoad^TM^/min, (E) acceleration distance > 1 m/s^2^/min, and (F) distance > 14 km/h/min, according to each discrete rating of perceived exertion (RPE) scores; a, b, c, d, e represent significant differences from RPE scores 1, 2, 3, 4, 5, respectively. The top of the bar is the median, and the error bars are the interquartile range.

Figure 1 shows the results of pairwise comparison of RPE values and intensity indicators. When analyzing internal training intensity (Avg HR %HR_max_ and Peak HR %HR_max_) through RPE values, Figures 1A and B demonstrate that broad comparisons between higher RPE ranges (RPE6–9) and lower ranges (RPE1–5) generally exhibited significant differences, whereas most adjacent comparisons within these ranges (e.g., RPE1 vs. RPE2, RPE7 vs. RPE8) showed no meaningful distinctions. For external training load variables, notable results emerged in specific contrasts. In Figure 1D (total PL/min), significant differences can be observed only between RPE4–9 and RPE1. In Figure 1E (Acc Dis > 1 m/s^2^/min), significant differences can be found exclusively between RPE6 and RPE1. In Figure 1F (Dis > 14 km/h/min), significant differences may be identified in the following comparative groups: RPE8 vs. RPE1, RPE5–9 vs. RPE3, and RPE7–9 vs. RPE2. We did not find any significant differences between RPE scores and Total Dis/min (Figure 1C). This pattern highlighted the discriminative capacity of the RPE in broader intensity bands rather than adjacent increments. Table 3 presents the detailed results of pairwise comparisons.

**Table 3:** Differences in internal (Avg HR %HR_max_, Peak HR %HR_max_) and external (Total PL/min, Acc Dis > 1 m/s^2^/min, Dis > 14 km/h/min) training intensity considering different RPE values.

Comparison (Median)	Effect size, r [95%CI]	*p*	Comparison (Median)	Effect size, r [95%CI]	*p*
The median of Avg HR (%HR_max_) across RPE 1–9 categories were: RPE 1 (61.88%), 2 (57.17%), 3 (62.75%), 4 (64.59%), 5 (64.81%), 6 (65.21%), 7 (65.85%), 8 (66.86%), 9 (65.50%)					
4–1	0.15 [0.08–0.22]	0.003	5–1	0.19 [0.12–0.26]	< 0.001
6–1	0.21 [0.13–0.28]	< 0.001	7–1	0.22 [0.15–0.29]	< 0.001
8–1	0.23 [0.16–0.30]	< 0.001	9–1	0.22 [0.15–0.29]	< 0.001
3–2	0.13 [0.06–0.21]	0.02	4–2	0.17 [0.09–0.24]	< 0.001
5–2	0.20 [0.13–0.27]	< 0.001	6–2	0.22 [0.14–0.29]	< 0.001
7–2	0.23 [0.16–0.30]	< 0.001	8–2	0.24 [0.17–0.31]	< 0.001
9–2	0.23 [0.16–0.30]	< 0.001	5–3	0.13 [0.06–0.21]	0.02
6–3	0.17 [0.09–0.24]	< 0.001	7–3	0.20 [0.12–0.27]	< 0.001
8–3	0.20 [0.13–0.27]	< 0.001	9–3	0.19 [0.11–0.26]	< 0.001
7–4	0.15 [0.08–0.23]	0.003	8–4	0.17 [0.09–0.24]	< 0.001
9–4	0.15 [0.08–0.22]	0.005			
The median of PeakHR (%HR_max_) across RPE 1–9 categories were: RPE 1 (87.23%), 2 (87.57%), 3 (88.66%), 4 (89.16%), 5 (91.96%), 6 (91.41%), 7 (91.46%), 8 (91.37%), 9 (89.03%)					
5–1	0.15 [0.07–0.22]	0.006	6–1	0.18 [0.10–0.25]	< 0.001
7–1	0.20 [0.12–0.27]	< 0.001	8–1	0.22 [0.15–0.29]	< 0.001
9–1	0.17 [0.09–0.24]	< 0.001	4–2	0.15 [0.07–0.22]	0.007
5–2	0.18 [0.11–0.25]	< 0.001	6–2	0.21 [0.14–0.28]	< 0.001
7–2	0.23 [0.15–0.30]	< 0.001	8–2	0.25 [0.18–0.32]	< 0.001
9–2	0.20 [0.13–0.27]	< 0.001	4–3	0.13 [0.05–0.20]	0.045
5–3	0.21 [0.13–0.28]	< 0.001	6–3	0.25 [0.18–0.32]	< 0.001
7–3	0.29 [0.22–0.35]	< 0.001	8–3	0.29 [0.22–0.36]	< 0.001
9–3	0.21 [0.13–0.28]	< 0.001	6–4	0.16 [0.08–0.23]	0.002
7–4	0.19 [0.12–0.26]	< 0.001	8–4	0.22 [0.14–0.29]	< 0.001
8–5	0.16 [0.09–0.23]	0.0013			
The median of Total PL/min across RPE 1–9 categories were: RPE 1 (5.41 AU/min), 2 (5.08 AU/min), 3 (6.09 AU/min), 4 (6.62 AU/min), 5 (7.13 AU/min), 6 (6.72 AU/min), 7 (6.56 AU/min), 8 (6.89 AU/min), 9 (6.70 AU/min)					
4–1	0.13 [0.05–0.20]	0.04	5–1	0.16 [0.09–0.24]	< 0.001
6–1	0.16 [0.08–0.23]	0.002	7–1	0.16 [0.09–0.23]	0.002
8–1	0.16 [0.08–0.23]	0.002	9–1	0.16 [0.09–0.23]	0.002
The median of Acc Dis > 1 m/s^2^/min across RPE 1–9 categories were: RPE 1 (8.25 m/min), 2 (7.91 m/min), 3 (9.49 m/min), 4 (10.30 m/min), 5 (10.00 m/min), 6 (10.10 m/min), 7 (10.00 m/min), 8 (9.03 m/min), 9 (9.78 m/min)					
6–1	0.13 [0.05–0.20]	0.041			
The median of Dis > 14km/h/min across RPE 1–9 categories were: RPE 1 (4.16 m/min), 2 (3.23 m/min), 3 (5.07 m/min), 4 (6.55 m/min), 5 (8.52 m/min), 6 (8.97 m/min), 7 (8.43 m/min), 8 (10.10 m/min), 9 (8.67 m/min)					
8–1	0.13 [0.05–0.20]	0.04	7–2	0.13 [0.06–0.20]	0.03
8–2	0.14 [0.07–0.22]	0.009	9–2	0.13 [0.05–0.20]	0.04
5–3	0.14 [0.07–0.21]	0.01	6–3	0.14 [0.07–0.22]	0.009
7–3	0.18 [0.10–0.25]	< 0.001	8–3	0.18 [0.11–0.25]	< 0.001
9–3	0.15 [0.07–0.22]	0.006			

RPE: rating of perceived exertion; Avg HR (%HR_max_): average heart rate expressed as a percentage of the maximum heart rate; Peak HR (%HR_max_): peak heart rate expressed as a percentage of the maximum heart rate; Total PL: total PlayerLoad^TM^; Dis > 14 km/h: running distance during speed threshold > 14 km/h; Acc Dis > 1 m/s^2^: accelerate distance during acceleration threshold > 1 m/s^2^. No significant differences were found between RPE scores in Total Dis/min

**Figure 2:**
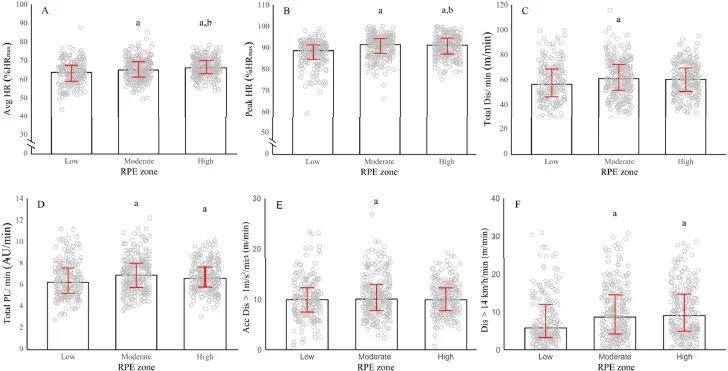
Distribution of (A) average HR (%HR_max_), (B) peak HR (%HR_max_), (C) total distance/min, (D) total PlayerLoad^TM^/min, (E) acceleration distance > 1 m/s^2^/min, and (F) distance > 14 km/h/min, according to the rating of perceived exertion (RPE) zone (Low: RPE 1–4, Moderate: RPE 5–6, High: RPE 7–10); a and b represent significant differences from low and moderate, respectively. The top of the bar is the median, and the error bars are the interquartile range.

Figure 2 shows the results of pairwise comparison of the RPE zone and intensity indicators. Significant differences were observed between particular RPE zones.

In Figure 2A and B (Avg HR %HR_max_ and Peak HR %HR_max_), significant differences can be observed in all pairwise comparisons among RPE zones. When the external load was considered (Total Dis/min, Total PL/min, Acc Dis > 1 m/s^2^ /min, Dis > 14 km/h /min), significant differences were observed between high- vs. low-intensity groups and between moderate- vs. low-intensity groups (Figure 2C–F). However, no significant differences were observed between the moderate group and the high group. Table 4 presents the detailed pairwise comparison results.

**Table 4:** Differences in internal (Avg HR %HR_max_, Peak HR %HR_max_) and external (Total Dis/min, Total PL/min, Dis > 14km/h/min, Acc Dis > 1 m/s^2^/min) training intensity under different RPE zones.

Comparison	Median, ES: r [95%CI], *p*	Comparison	Median, ES: r [95%CI], *p*
Avg HR (%HR_max_), %		Peak HR (%HR_max_), %	
Low-High	63.81–65.09, 0.26 [0.19–0.33], < 0.001	Low-High	88.67–91.24, 0.31 [0.24–0.38], < 0.001
Moderate-High	65.09–66.19, 0.11 [0.03–0.18], 0.02	Moderate-High	91.46–91.24, 0.12 [0.05–0.19], < 0.001
Moderate-Low	65.09–63.81, 0.20 [0.13–0.27], < 0.001	Moderate-Low	91.46–88.67, 0.25 [0.18–0.32], < 0.001
Total Dis/min, m/min		Total PL/min, AU/min	
Low-High	56.70–60.39, 0.08 [0.01–0.16], > 0.05	Low-High	6.24–6.61, 0.15 [0.07–0.22], < 0.001
Moderate-High	61.09–60.39, 0.02 [−0.05–0.1], > 0.05	Moderate-High	6.90–6.61, 0.02 [−0.06–0.09], > 0.05
Moderate-Low	61.09–56.70, 0.11 [0.04–0.19], 0.0101	Moderate-Low	6.90–6.24, 0.15 [0.08–0.23], < 0.001
Acc Dis > 1 m/s^2^/min, m/min		Dis > 14km/h/min, m/min	
Low-High	9.92–9.92, 0.07 [−0.01–0.14], > 0.05	Low-High	5.77–9.02, 0.19 [0.11–0.26], < 0.001
Moderate-High	10.10–9.92, 0.02 [−0.05–0.1], > 0.05	Moderate-High	8.65–9.02, 0.08 [0.00–0.15], > 0.05
Moderate-Low	10.10–9.92, 0.10 [0.02–0.17], 0.03	Moderate-Low	8.65–5.77, 0.14 [0.06–0.21], 0.001

RPE: rating of perceived exertion; Avg HR (%HR_max_): average heart rate expressed as a percentage of the maximum heart rate; Peak HR (%HR_max_): peak heart rate expressed as a percentage of the maximum heart rate; Total Dis: total running distance; Total PL: total PlayerLoad^TM^; Acc Dis > 1 m/s^2^: accelerate distance during acceleration threshold > 1 m/s^2^; Dis > 14 km/h: running distance during speed threshold > 14 km/h

## Discussion

The aims of this study were to (1) investigate the association between subjective perception (RPE, sRPE) and objective metrics (TRIMP, average HR and peak HR both expressed as %HR_max_, total distance, PlayerLoad^TM^, acceleration distance > 1 m/s^2^, and running distance ≥14 km/h), and (2) examine the relationship between two methods of the RPE use (RPE values and RPE zones) and training intensity response in collegiate male soccer players. Based on the magnitude of repeated measurement correlations (Table 2), our study found that the sRPE had a moderate to large significant correlation with TRIMP1, TRIMP2, PlayerLoad^TM^, total distance covered, distance covered at > 14 km/h and acceleration distance (> 1 m/s^2^), with r ranges from 0.34 to 0.60 (*p* < 0.001). In contrast, the correlation between the RPE and intensity metrics was weaker, with only small to moderate relationships observed (r ranges from 0.11 to 0.35, *p* < 0.01). When the RPE and RPE zones were used to classify training intensity, significant differences were found in various intensity metrics. However, significant differences were not detected in all pairwise comparisons (Figures 1 and 2). The utility of categorizing external/internal training intensity requires consideration of athlete-reported specific RPE values.

The association between internal and external measurements of training loads and intensity is crucial for understanding the dose-response relationship in team sport training (McLaren et al., 2018). In soccer, the primary methods for evaluating internal loads include a subjective approach based on the sRPE and an objective approach based on heart rate metrics (e.g., TRIMP or time spent in heart rate zones) (Helwig et al., 2023). Our findings align with previous research on Australian football (Bartlett et al., 2017; Gallo et al., 2015) and soccer players (Casamichana et al., 2013; Gaudino et al., 2015; Scott et al., 2013), showing that the sRPE has moderate to strong correlations with total distance covered, PL, distance covered in different speed zones, and accelerations. Additionally, the high correlation between the sRPE and training impulse (TRIMP) supports the sRPE as an effective and widely used method for measuring the internal load (Alexiou and Coutts, 2008; Casamichana et al., 2013; Costa et al., 2022; Impellizzeri et al., 2004). In running-based sports like soccer, the close relationship between the sRPE and training loads is reasonable as, from a physiological perspective, longer distances and faster speeds require increased energy metabolism (Wallace et al., 2014). In the process of the body driving muscle contraction through energy metabolism, carbohydrates, fats, proteins, and aerobic energy consumption are required to provide oxygen (Vanrenterghem et al., 2017). Such a process mainly involves cardiopulmonary function, thus the internal load is usually related to cardiac output and oxygen uptake (Vanrenterghem et al., 2017). Furthermore, the neural discharge process driven by central motor commands to the lower limb and respiratory muscles is theoretically believed to contribute to the perception of effort (Marcora, 2009). Regarding the relationship between the internal and the external load, among the sRPE, heart rate based indices, and external load metrics (i.e., distance, player load), the external load induces the internal load (sRPE and heart rate based indices) (Helwig et al., 2023). However, RPE values reported by players are influenced by multiple factors, including the perception of respiratory muscle effort (which may be reflected in heart rate metrics), hormonal responses, and blood lactate levels, and other contributing factors that may potentially encompass participants’ characteristics and environmental conditions (De Dios-Álvarez et al., 2024; Haddad et al., 2017). As a result, heart rate-based methods are often considered limited because they do not account for anaerobic metabolism during exercise (Alexiou and Coutts, 2008; Borresen and Ian Lambert, 2009). In contrast, subjective methods such as the RPE are viewed as a more holistic approach, as they incorporate multiple psychophysiological factors. A previous meta-analysis also indicated that, in running activities primarily powered by aerobic metabolism, the sRPE showed a stronger correlation with total distance compared to heart rate-based metrics (such as TRIMP) (McLaren et al., 2018). However, the absence of a “perfect” relationship between the sRPE and other internal and external load metrics is also reasonable, given the multiple factors that need to be considered. Despite this high correlation, the high (> 10%) intraindividual and interindividual variability of the sRPE still warrants attention, and practitioners may need to pay more attention to individual variations in specific players. It is important to note that our study used generalized speed thresholds, while individualized speed thresholds may be more suitable for the characteristics of the study population and tend to show an increase in correlation (Rago et al., 2019).

When using the sRPE to assess training loads, the RPE is often considered to assess the intensity of training. The existing evidence suggests weak or indeterminate associations between the RPE and time-adjusted intensity metrics, including distance, PL, acceleration, impact events, and high-speed running distance—all normalized to per-minute values (Gaudino et al., 2015; Marynowicz et al., 2020; Rago et al., 2022). When using discrete RPE or RPE values, to differentiate between internal training intensities (average HR %HR_max_, peak HR %HR_max_), there appears to be significant differences between different RPE values (Figure 1). In the study by Costa et al. (2022), each discrete RPE score corresponded to the value of the average HR (%HR_max_), and showed a gradually increasing trend (RPE 2, 68%, 65–69%; RPE 3, 72%, 69–75%; RPE 4, 79%, 76–82%; RPE 5, 84, 81–87%, median and interquartile intervals). In this study, we did not find an obvious step-by-step correspondence between the RPE and peak HR %HR_max_, and at higher RPE values, although the Bonferroni test results did not show significance, the higher RPE value (RPE = 9) corresponded to smaller average HR %HR_max_ and peak HR %HR_max_ (median, 65.50% and 89.03%, respectively), compared to the lower value (RPE = 8) (median, 66.85% and 91.37%, respectively). This is similar to previous research in professional ice hockey by Rago et al. (2022), which showed that a higher RPE value (RPE = 7) corresponded to a smaller median of the HR mean (%HR_max_) and the peak HR (%HR_max_) than a lower RPE value (RPE = 6). In fact, during higher intensity exercise, the proportion of anaerobic energy supply increases. Heart rate and heart rate- related variable assessments of the internal load appear to ignore the contribution of blood/muscle lactate (Bangsbo et al., 2008; Barron et al., 2014). In the study by Costa et al. (2022) about technical training sessions, the range of the RPE was only limited to 2–5, while according to the study by Seiler et al. (2006) in endurance athletes, the range between 4 and 5 is the breakpoint of the first ventilatory threshold (VT1), and between 6 and 7 is the breakpoint of the second ventilatory threshold (VT2). The range of the RPE of 2–5 is mainly based on aerobic energy supply, and heart rate-based assessment seems to be suitable for this scenario. When the RPE value is higher, it seems that more factors including lactate need to be considered. Based on the research of Seiler et al. (2006), we used RPE zones to divide training intensity (Figure 3). Our research shows that RPE zones are also an effective way to divide training intensity, and significant differences were found in each zone in the average HR %HR_max_ and peak HR %HR_max_.

The use of the RPE to divide external training load intensity appears to be extremely limited. Under the total distance per minute, no significant differences were found among RPE1 to RPE9. In contrast, under the PL per minute, RPE4 to RPE9 each showed significant differences compared to RPE1. In acceleration distance (> 1 m/s^2^) per minute, only RPE 6 showed significant differences with RPE 1. In distance covered at speed > 14 km/h per minute, there were significant differences between multiple high and low RPE values (Figure 1 and Table 3).

In terms of RPE zones, under the total distance per minute, a significant difference was observed between moderate and low RPE categories. Under the total PL per minute, significant differences were found between moderate and low RPE categories, as well as between high and low RPE categories. While no significant differences were found when the RPE was analyzed at individual levels (Figure 1C), grouping RPEs into zones revealed significant differences under the total distance per minute metric (Figure 2C). This may be due to the increased sample size within each zone and the more pronounced contrast between broader categories, which potentially enhances the ability to detect meaningful differences. When considering other metrics, moderate and high RPEs showed significant differences only compared to the low RPE, while there was no significant difference between moderate and high RPEs.

## Conclusions

Our study provides evidence for understanding the relationship between the subjective load assessment method (sRPE) and objective methods. The sRPE showed a moderate to large correlation with load volume metrics, while the RPE had a weaker correlation with load intensity. High intra- and inter-individual variability should be considered to understand individual athlete’s responses. Based on the range of the RPE of 1–9, specific RPE values and RPE zones appear to significantly distinguish between internal and external training intensity, with a seemingly better effect on internal intensity compared to external intensity.
